# The Daily Mile Is Able to Improve Cardiorespiratory Fitness When Practiced Three Times a Week

**DOI:** 10.3390/ijerph17062095

**Published:** 2020-03-22

**Authors:** Paolo Riccardo Brustio, Anna Mulasso, Corrado Lupo, Alberto Massasso, Alberto Rainoldi, Gennaro Boccia

**Affiliations:** 1 NeuroMuscular Function Research Group, School of Exercise and Sport Sciences, Department of Medical Sciences, University of Turin, 10143 Turin, Italy; paoloriccardo.brustio@unito.it (P.R.B.); anna.mulasso@unito.it (A.M.); corrado.lupo@unito.it (C.L.); gennaro.boccia@unito.it (G.B.); 2 Dipartimento di Prevenzione dell’ASL TO 4, 10072 Caselle Torinese, Italy; amassasso@aslto4.piemonte.it

**Keywords:** dose–response, school-based physical activity, intervention, active break

## Abstract

The Daily Mile is a promising initiative aimed at removing some of the barriers to physical activity in the school setting. This quasi-experimental study investigated the dose–effect of The Daily Mile on cardiorespiratory fitness, waist-to-height ratio, and body mass index (BMI) after a period of 3- and 6-months. A total of 279 students (mean age = 9 ± 1 years) participated in The Daily Mile while 269 students (mean age = 9 ± 1 years) did not (control group). A posteriori, the classes performing The Daily Mile on average two times per week were included in the 2_times subgroup, while those performing the activity on average three times per week in the 3_times subgroup. A significant difference was observed in favor of the experimental compared to the control group in the 6 Minute Run Test (F = 13.932, *p* = 0.008). Moreover, the improvement of the 6-minute run test was more pronounced for 3_times (effect size = 0.51) rather than for the 2_times subgroup (effect size = 0.29). No differences were observed in waist-to-height ratio and BMI scores. In conclusion, teachers are strongly recommended to implement The Daily Mile at least three times a week to see appreciable effects on cardiorespiratory fitness.

## 1. Introduction

Regular physical activity is considered a milestone for health promotion and wellbeing, especially throughout childhood and adolescence [1,2]. International guidelines point out that children and adolescences (aged 5–17) should perform at least 60 min of moderate- to vigorous-intensity physical activity (MVPA) per day [3,4] to prevent and/or reduce the incidence of overweight [5,6] and non-communicable diseases in adulthood [7]. Importantly, one out of the nine global targets stated by the World Health Organization for improving the prevention and treatment of non-communicable diseases is to reduce the prevalence of insufficient physical activity by 10% by 2025 [3,8].

Despite this, compliance with MVPA recommendations for European children is generally low [9]. Independently from gender and country, the prevalence of children (aged 2–10 years) performing at least 60 min daily of MVPA ranges from 23.2% to 50.8%. In Italy, these trends are even more alarming, recently highlighting that only about 1 child out of 10 meets MVPA recommendations appropriately [10]. Low physical activity levels in childhood are predictive of overweight and obesity, therefore, 35.2% of Italian children aged 7–13 years are classified as overweight or obese and 12.2% are classified as obese [11].

To better promote children’s welfare, different strategies, such as the introduction of short bouts of physical activity, have been implemented in the Italian school context [12,13]. The Daily Mile^TM^ is a promising initiative aimed at increasing MVPA by removing some of the barriers to be physically active within the school setting [14]. The Daily Mile activity consists of running or jogging for 15 min (approximately one mile) at their own pace, outside the school buildings. The intervention is conducted by teachers, does not require any specific equipment, and allows teachers the autonomy to carry out the initiative [15,16].

The activity is successfully implemented and accepted within the school day schedule of primary schools in different educational contexts [15,16,17]. Given the simple intervention characteristics (e.g., the short execution time and relatively low cost) and the flexible organization that supports the teacher’s autonomy, The Daily Mile appears to be acceptable and feasible [18]. Teachers recognize the potential of such an intervention to improve cardiorespiratory fitness [19] and to reduce negative health consequences (such as childhood overweight) [17,20]. Moreover, The Daily Mile offers social time to facilitate/promote the relationships between peers and teachers [17,20] and supportive school climate [21].

Previous studies suggested that The Daily Mile was effective at increasing cardiorespiratory fitness after three [15] and six months of activity, as well as at improving body composition in the primary school context [16]. Furthermore, the implementation of The Daily Mile results in a relative increase of approximately 9 min per day in MVPA together with a decrease of approximately 18 min per day in sedentary behavior over time [16].

In consideration of all the above-mentioned findings, it could be underlined that The Daily Mile may be considered a public health intervention to promote wellbeing throughout childhood. However, no previous studies have investigated the dose–response effect of The Daily Mile on cardiorespiratory fitness and body composition. Indeed, even though it is suggested to perform the activity at least 3 times a week [14], no empirical evidence supports this finding. Furthermore, as in school settings, the amount of time allocated to physical activity is limited; it is necessary to understand the minimum weekly dose to observe fitness changes. Thus, this study aimed to investigate the dose–effect of The Daily Mile on cardiorespiratory fitness (primary outcome), and waist-to-height ratio and body mass index (BMI; secondary outcomes) after a period of three and six months. Specifically, we investigated how the weekly frequency of executing The Daily Mile may impact on children’s cardiorespiratory fitness and their waist-to-height ratio and BMI.

## 2. Materials and Methods

### 2.1. Design

A quasi-experimental study was conducted using a between-subjects design over 6 months. Two convenience samples of school classes were included in the experimental and control group based on voluntary adhesion to perform The Daily Mile. The experimental group agreed to include The Daily Mile program in the school day routine across a period of six months, while the control group decided not to participate and to continue the usual daily school activity. A posteriori, the experimental group was furthermore divided into two subgroups: (1) *3_times* group, i.e., those classes that performed The Daily Mile as per its core principles more than 2.5 times a week on average; (2) *2_times* group, i.e., those classes that performed The Daily Mile less than 2.5 times a week on average. Therefore, the sample was composed of three groups: two experimental subgroups (i.e., 3_times and 2_times) and a control group. The primary outcome of the study was the assessment of cardiorespiratory fitness via the performance in the 6-min run test. The secondary outcomes were the waist-to-height ratio and BMI. The participants were assessed three times: at baseline (pre-test); after 3 months from the beginning of the study (mid-test); after 6 months, (i.e., post-test)

### 2.2. Participants

In September 2018, we organized (in the neighborhood of Turin, Italy) a public meeting open to primary school teachers to make them aware of the existence of The Daily Mile and the organization of this study. During this information session, we delivered a 30-min talk presenting organizational details of the study, provided basic advice to implement The Daily Mile, and invited teachers to visit The Daily Mile web site [14] for more details. After that, teachers independently decided to take part in the study and to introduce or not The Daily Mile in their classes. All schools/classes recruited for the study were part of the same institutional and regional office and therefore were receiving a similar delivery of educational curricula.

Parents/guardians and teachers provided written informed consent for participation in the study, according to the ethical standards provided in the 1964 Declaration of Helsinki. Additionally, a student’s consent form, written in clear and simple language, was included. The study was approved by the Ethical Committee of the University of Torino (protocol number: 203427).

### 2.3. Intervention: The Daily Mile

The Daily Mile requires children to run, jog, or walk at their own pace for 15 min (~one mile) outside the school building during the school time. The emphasis of the activity is on the time, not on the distance [14]. Indeed, children may cover more or less than one mile during the activity. The activity was conducted by the teachers that could choose when to perform the intervention (e.g., when it best suited to their timetable). According to The Daily Mile guidelines [14], teachers were instructed to perform the activity as a break lasting 15 min from when the pupils get up from the school desk to when they return to sit. Children performed the activity with their normal school clothes. The activity did not replace normal physical education lessons and/or breaks. Children with an intellectual or physical disability fully participated in the activity and the data collection. If they were not able to properly execute the 6-min run test, their data were not included in the analysis. The teachers weekly monitored the adherence of their class to the program by annotating on a booklet each occasion the class went out to execute The Daily Mile.

### 2.4. Assessment

Pre-, mid-, and post-test assessments of the height, weight, waist circumstance, and 6-min run test were performed at the schools’ gym by the same trained and qualified investigators. The children underwent the measurements according to a random order within a single session lasting 1–2 h according to class size and school timetable.

Height and weight were measured using a portable stadiometer (Model 214; Seca, Hamburg, Germany) with an accuracy of 0.01 m, and an electronic scale (Model 876; Seca, Hamburg, Germany) with an accuracy of 0.1 kg, respectively. Waist circumstance was measured to the nearest 0.01 m in the midway between the lowest rib and the iliac crest with the pupils in standing position. The waist-to-height ratio was calculated by dividing waist circumference by height. The BMI was calculated as body mass divided by height squared (kg·m^−2^).

The 6-min run test requires pupils to run as far as possible for 6 min and it was used to assess cardiorespiratory fitness [22,23]. Students were instructed to run on a circuit in the gym and the distance covered by each participant was measured in meters. The test had an intraclass correlation coefficient of 0.86 in children aged 6–10 years [22].

### 2.5. Statistical Analyses

Socio-demographic and baseline physical characteristics were compared among experimental and control groups using independent analysis of variance (quantitative variables) or chi-squared test (qualitative variables) and presented as the mean or percentage frequency (95% CI).

The classes in the experimental group were subgrouped a posteriori based on the average weekly frequency they performed The Daily Mile. We adopted a threshold of 2.5 times per week: the classes that executed The Daily Mile less than 2.5 times per week on average were included in the 2_times subgroup, while those who executed The Daily Mile more than 2.5 times per week were included in the 3_times subgroup.

Using as covariates the values of age and BMI at pre-test, a series of repeated-measures ANOVA, with group (i.e., 2_times, 3_times, and control) and gender (i.e., female and male) as between-subject factors, and time (i.e., pre-, mid-, and post-test) as within-subject factor, was performed for 6-min run test and waist-to-height ratio outcomes. Differently, using age as covariates, a repeated-measures ANOVA, with group and gender as between-subject factors and time as within-subject factor, was performed for BMI outcome.

Differences between the experimental and control groups were determined by significant group × time interactions. Moreover, group × gender × time interactions were evaluated to investigate possible gender differences in the three different groups during the time. The effect size was determined using partial η^2^. A post hoc analysis with a Bonferroni adjustment was computed to identify statistically significant interactions. Differences between pre- vs. mid-, mid- vs. post-, and pre- vs. post-test were reported in percent values (calculated on raw data) the precision of estimates for absolute values was indicated with 95% confidence interval (CI). The magnitude of the difference was calculated as Cohen’s d effect size (95% CI). Threshold values for effect size statistics were: >0.2, small; >0.5, medium; >0.8, large [24]. The level of significance was set at *p* = 0.05. The Statistical Package for Social Sciences (SPSS Inc., version 25.0 for Windows, Chicago, IL, USA) and statistical software GraphPad Prism 8.0 (San Diego, California, USA) were used for all statistical analyses.

## 3. Results

A total of 17 classes, including 279 students (mean age = 9 ± 1 years; 46.8% female), composed the experimental group. Specifically, 12 classes, involving 168 students (mean age = 9 ± 1 years; 49.1% female), were included a posteriori in the 2_times subgroup, whereas 5 classes, involving 111 students (mean age = 9 ± 1 years; 50.6% female), were included in the 3_times subgroup (20.3%) based on how many times a week, on average, they performed The Daily Mile. All classes completed the 6-months intervention in their allocation group, i.e., no drop out was registered. The 2_times group performed The Daily Mile on average two times a week, while the 3_times group performed The Daily Mile three times per week. Conversely, a total of 15 classes, including 269 students (mean age = 9 ± 1 years; 49.1% female) did not participate in The Daily Mile and composed the control group.

Table 1 reports the detailed socio-demographic data and baseline physical data of the sample. No difference was observed among groups in terms of gender, weight, waist circumstance, waist-to-height ratio, and BMI (all *p* > 0.05). Unfortunately, some differences were observed at baseline in terms of age (F = 9.924; *p* < 0.001), height (F = 5.391; *p* = 0.005), and 6-min run test performance (F = 10.333; *p* < 0.001).

Table 2 shows the raw data (not corrected for covariates) of the primary and secondary outcome measures for pre-, mid-, and post-test assessments and repeated ANOVA outcomes.

**Figure 1:**
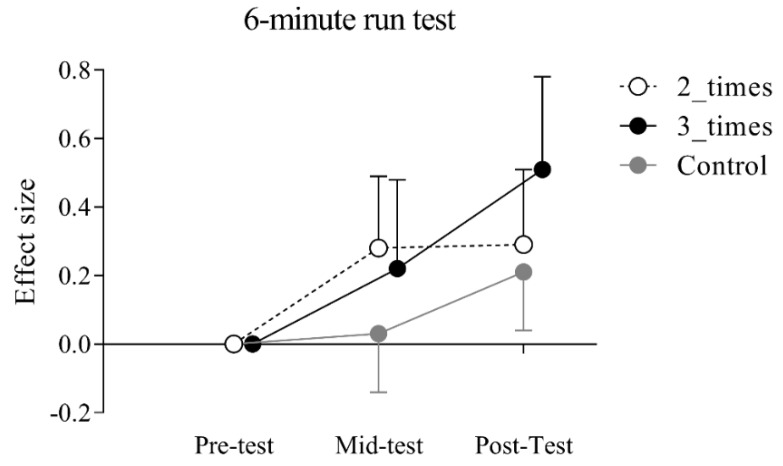
Mean effect size and 95% confidence interval (CI) changes in cardiorespiratory fitness in the three different groups.

After correcting for age and BMI at pre-test, no significant group × gender × time interaction was observed in the primary outcome, i.e., the performance in the 6-min run (F = 1.004, partial η^2^ = 0.003, *p* = 0.404). Differently, significant group × time interaction (F = 3.356, partial η^2^ = 0.009, *p* = 0.009) was observed. In the 2_times group, post-hoc analysis with Bonferroni adjustment revealed an overall statistically significant higher score between pre- and mid-test [estimated mean difference = 33.29, 95% CI (3.06, 63.51) m; *p* = 0.015; effect size (ES) = 0.28] and between pre- and post-test [estimated mean difference = 35.64 95% CI (5.42, 65.87) m; *p* = 0.005; ES = 0.29], but not between mid- and post-test (*p* = 1.00). In 3_times group, post-hoc analysis with Bonferroni adjustment showed an overall statistically significantly higher score between mid- and post-test (estimated mean difference = 42.99, 95% CI (5.80, 80.17) m; *p* = 0.07; ES = 0.32] and between pre- and post-test (estimated mean difference = 69.59, 95% CI (32.46, 106.73) m; *p* < 0.001; ES = 0.51], but not between pre- and mid-test (*p* = 0.792). Differently, no significant differences were observed in the control group between pre- and mid-test (*p* = 1.00), but only between mid- and post-test [estimated mean difference = 30.63, 95% CI (6.74, 54.52) m; *p* < 0.01; ES = 0.18] and between pre- and post-test [estimated mean difference = 36.50, 95% CI (12.64, 60.38) m; *p* < 0.01; ES = 0.21]. Figure 1 provides the effect size changes between pre-, mid-, and post-test separately for each group.

Repeated measures ANOVA outcomes showed no significant group × gender × time interactions were observed (F = 0.463, partial η^2^ = 0.002, *p* = 0.762) while significant group × time interaction was observed in the waist-to-height ratio (F = 6.340, partial η^2^ = 0.022, *p* < 0.001). No significant differences over the time were observed in 2_times and control groups (all *p* > 0.05). Differently, in 3_times post-hoc analysis with Bonferroni adjustment showed an overall significant lower score between pre- and mid-test [estimated mean difference = −0.014, 95% CI (−0.024, −0.003); *p* = 0.01; ES = −0.26], but not between mid-test and post-test and between pre-test and post-test (*p* = 0.813).

Finally, no significant group × gender × time interactions (F = 1.393, partial η^2^ = 0.005, *p* = 0.234) and group × time interactions were observed in BMI (F = 1.280, partial η^2^ = 0.004, *p* = 0.275).

## 4. Discussion

The present quasi-experimental study investigated how the weekly frequency of executing The Daily Mile may impact children’s fitness in the Italian school context. The main finding of our study was that, over six months, executing The Daily Mile on average three times a week confers more beneficial effects on the 6-min run test performance compared to an average execution of two times a week. Executing the activity two times a week provided some improvement in the first three months of the study, but then did not provide any further improvement. The present study did not find any difference in terms of BMI and waist-to-height ratio.

The experimental group, when considered as a whole (i.e., 2_times and 3_times subgroups merged), showed an improvement in cardiorespiratory fitness over six months, independently of gender. Indeed, the pupils engaged in The Daily Mile showed an increase in the performance of the 6-min run test of 49 m, corresponding to an improvement of 7.2 % (based on raw data). Using a similar research setting in the Italian context, but with a different length of intervention (i.e., 3 months), we previously showed an improvement for the 6-min run test by 7.5% (estimate adjusted for age and gender: 3.1% increase) [15]. Similarly, in Scotland, Chesham et al. (2018) found an improvement of 0.23 SMD (standardized mean difference) in the shuttle run after 8 months of engagement in The Daily Mile. Therefore, the present results corroborated previous research on the efficacy of The Daily Mile in ameliorating the physical fitness of children. To our knowledge, no previous study investigated the dose–response effect between the weekly frequency of engagement in The Daily Mile and cardiorespiratory fitness. Here we found that the frequency of execution may impact children’s physical functions. Pupils engaged in the Daily Mile were divided into two different groups: one group that performed the activity at least 2 times a week (i.e., 30 min of activity per week) and one group that performed the activity at least three times a week (i.e., 45 min of activity per week). Both groups showed improvements in the 6-min run test at the end of the intervention. However, considering pre- and post-test assessment, we observed a more pronounced improvement in 6-min run test for students engaged in The Daily Mile on average three times a week (ES = 0.51, raw percentage increase 8.8%) compared to those with a frequency of two times a week (ES = 0.29, raw percentage increase 5.6%). This finding points out that The Daily Mile may improve cardiorespiratory functions in a dose–response fashion (See Figure 1).

In general, the improvement found in the present study was smaller than expected. For example, the group that performed The Daily Mile two times a week improved similarly to the control group. The improvement of 6-min run test performance in the control group was expected since the children were in a phase of biological maturation. However, we expected to find a larger improvement in those doing The Daily Mile two times a week, despite being smaller in magnitude compared to those executing it three times a week as indicated in the core principles [14]. Moreover, the improvement found in the group that performed The Daily Mile three times a week, which lasted about six months, was only slightly larger (raw percentage increase of 8.8% on average) of that found in the previous study [15] lasting only three months (raw percentage increase of 7.2 on average). Beyond the inter-subject variability in responses to cardiorespiratory training, many other experimental variables, which were not controlled in this study, may have affected these observations. First, the effort that each child put in the execution of the activity may affect the outcome variables. Since we did not quantify the pace or distance performed each day by the pupils, we were not able to control this variable. Second, in the period of the school year investigated in the study, the Italian schools observed several religious and lay holidays (for instance, Christmas, Carnival, Easter, International Workers’ Day). Since it is known that the consistency in executing physical activity is a key to experience tangible appreciable improvement, the interruptions in the daily scholastic activities may have interfered with the beneficial effect of The Daily Mile. Third, the seasonal variation in terms of climate or weather might have influenced the compliance and intensity of activity. Fourth, when interpreting results, we need to consider the baseline difference among groups (see Table 1). Since we could not randomize the classes participating in the program, differences at baseline may be expected. Because of this, the higher value observed at baseline in the 3_times group compared to 2_times and control groups would have made any effects of the intervention harder to observe. All the above-mentioned aspects should be taken into account when interpreting our results.

A small but significant reduction by 2.7% emerged after 3 months of activity The Daily Mile three times a week independent of the gender. This improvement in waist-to-height ratio obtained in the 3_times is noteworthy because lower scores in the waist-to-height ratio are associated with decreased risk for central obesity [24], metabolic syndrome, pre-diabetes, hypertension, and dyslipidemia [25,26]. Unfortunately, three months later, that is, 6 months after the beginning of the intervention, the waist-to-height ratio came back near to baseline values. In the Scottish Daily Mile, the authors observed a reduction in adiposity measured by skinfolds 8 months after the introduction of the activity [16]. Conversely, our previous study on The Daily Mile efficacy in the Italian context did not find any difference in waist-to-height ratio after three months of activity [15]. Accordingly, no improvement in BMI was observed in this study, even when considering the higher weekly frequency. Some criticism and skepticism for public health perspective arise on the effect of The Daily Mile, regarding the effect on BMI and overweight [27]. Although the acute [28] and long-term effects [16] of increasing MVPA were previously demonstrated (about an increase of one-third of the daily recommendations), few pieces of evidence have shown an effect on body weight reduction probably due to the limited impact of 15 min of activity per day [17]. This is in accordance with the finding of a previous meta-analysis showing no improvement in BMI after school-based physical interventions lasting for a minimum of 6 months [29]. It may be possible, that the length of the intervention, being as short as six months, may not be long enough to improve BMI. Indeed, it has been demonstrated that only long-term school-based physical interventions, ranging from 12 to 72 months, are able to improve BMI [30]. Moreover, it has been well established that multicomponent programs (including physical activity intervention, parent training, behavioral, dietary, and nutrition education) rather than only physical activity interventions seem to be more effective to counteract overweight and obesity in childhood [31].

The present study had some relevant limitations to be underlined. Although this is the first study assessing the dose–response effect between The Daily Mile engagement and physical fitness, it only considers the frequency of two or three times a week. Future studies investigating a wider range of frequency (ideally from two to five times a week) are needed to better delineate the effect of weekly frequency on physical outcomes. Additionally, we considered only cardiorespiratory fitness, waist-to-height ratio and BMI as primary and secondary outcomes of our study. Future studies should investigate the feasibility and potential dose–response effect of The Daily Mile on increasing MVPA. Despite all the schools involved in the study were part of the same institutional and regional office and therefore were receiving similar delivery of educational curricula, we were not able to discriminate our sample on the base of socio-economic characteristics. Thus, we cannot exclude that socio-economic status may have affected the results of the present study [16]. Moreover, it is plausible that those teachers who voluntarily decided to introduce The Daily Mile in their classes (experimental group) were particularly sensitive to the topic of physical activity compared to those who decided to not implement the program. These differences may have affected the results and may have limited the representativeness of the samples in our study. Therefore, our results should be interpreted with caution. Future research, using a cluster randomized controlled trial, a wider range of schools and age is needed to better generalize our results in Italian educational contexts. Furthermore, within the same schools, some classes belonged to the experimental and some other to the control groups. Thus, there was a possible contamination effect between experimental and control groups. Despite these aspects representing methodological limitations, we believe that this also represents a “real world” setting to better understand the effects of the Daily Mile on public health.

## 5. Conclusions

Identifying the potential dose–response effect between The Daily Mile engagement and physical functions in children would be important to optimize the implementation of The Daily Mile. The present study investigated the effects of weekly frequency of The Daily Mile on cardiorespiratory fitness, BMI, and waist-to-height ratio. Considering the unstructured and ecological nature of the activity, our data suggest that executing The Daily Mile on average 3 times a week was more beneficial to children’s fitness than two times a week. Executing the activity two times a week conveyed a benefit in the first three months but did not provide further improvement later. Thus, after six months, it did not seem to provide a beneficial effect compared to the control condition. Therefore, to see appreciable effects on physical fitness, teachers are strongly recommended to implement the activity at least three times a week.

## Figures and Tables

**Table 1:** Socio-demographic and physical characteristics of the study participants. Data are reported as frequency or mean and (95% CI).

Variables	All (*n* = 548)	2_times (*n* = 168)	3_times (*n* = 111)	Control (*n* = 269)	*p* Value
Female	49.1 (44.8, 53.4)	50.6 (42.8, 58.4)	46.8 (37.30, 56.6)	49.1 (42.9, 55.2)	*p* = 0.829
Male	50.9(46.6, 55.2)	49.4(41.6, 57.2)	53.2 (43.4, 62.7)	50.9 (44.8, 57.1)	
Age (years)	9.14 (9.07, 9.21)	8.91 (8.78, 9.04) ^a,b^	9.19 (9.05, 9.33) ^b^	9.26 (9.16,9.35)	*p* < 0.001
Weight (kg)	34.65 (33.96, 35.34)	33.99 (32.70, 35.28)	34.20 (32.66, 35.73)	35.25 (34.27, 36.22)	*p* = 0.244
Height (m)	1.36 (1.35, 1.37)	1.35 (1.34, 1.36) ^a^	1.36 (1.35, 1.37) ^b^	1.37 (1.36, 1.38)	*p* = 0.005
Waist circumstance	64.56 (63.87, 35.26)	64.15 (62.84, 65.46)	63.49 (62.03, 64.95)	65.27 (64.27, 66.26)	*p* = 0.120
6-min run test (m)	823 (810, 835)	781 (763, 800) ^a^	860 (838, 883) ^b^	833 (812, 854)	*p* < 0.001
Waist-to-height ratio	0.471 (0.467, 0.476)	0.474 (0.466, 0.482)	0.465 (0.456, 0.474)	0.47 (0.47, 0.48)	*p* = 0.289
BMI (kg·m^−2^)	19.3 (18.0, 20.6)	20.0 (16.9, 23.1)	20.3 (16.3, 24.2)	18.4 (18.0, 18.8)	*p* = 0.397

*Notes:*^a^, significant difference from 3_times; ^b^, significant difference from control group.

**Table 2:** Percentage difference from pre-, mid-, and post-test and repeated measure outcomes.

Variables	PRE vs. MID		MID vs. POST		PRE vs. POST	
	% Δ	ES	% Δ	ES	% Δ	ES
**2_times**						
6-min run test (m)	5.2 (3.3, 7.1) *	0.28, S	0.9 (−0.8, 2.6)	0.02, T	5.6 (3.6, 7.6) *	0.29, S
Waist-to-height ratio	1.7 (0.8, 2.6)	0.13, T	−1.2 (−2.1, −0.3)	−0.13, T	0.3 (−0.6, 1.2)	0.00, T
BMI (kg·m^−2^)	1.9 (0.3, 3.5)	0.07, T	0.2 (−1.2, 1.6)	−0.01, T	1.7 (0.1, 3.3)	0.06, T
**3_times**						
6-min run test (m)	3.6 (1.7, 5.5) *	0.22, S	5.5 (2.8, 8.2) *	0.32, S	8.8 (6.0, 11.6) *	0.51, M
Waist-to-height ratio	−2.7 (−3.8, −1.6) *	−0.26, S	1.6 (0.0, 2.3)	0.13, T	−1.2 (−2.6, 0.2)	−0.11, T
BMI (kg·m^−2^)	−1.4 (−2.7, −0.1)	−0.10, T	2.4 (1.1, 3.7)	0.12, T	0.7 (−0.7, 2.1)	0.02, T
**Control**						
6-min run test (m)	2.9 (0.5, 5.3)	0.03, T	5.1 (3.0, 7.2) *	0.18, T	6.2 (4.1, 8.3) *	0.21, S
Waist-to-height ratio	−0.5 (−1.4, 0.4)	−0.07, T	1.2 (0.2, 2.2)	0.08, T	0.4 (−0.6, 1.4)	0.00, T
BMI (kg·m^−2^)	2.4 (1.4, 3.4)	0.00, T	2.3 (0.8, 3.8)	0.20, T	4.2 (3.0, 5.4)	0.22, S

Note: *, significant difference from comparison. Δ percentage difference; ES, effect size magnitude: T, trivial; S, small; M, medium.
